# Compensatory mechanisms of reduced interhemispheric EEG connectivity during sleep in patients with apnea

**DOI:** 10.1038/s41598-023-35376-1

**Published:** 2023-05-25

**Authors:** Maksim Zhuravlev, Mikhail Agaltsov, Anton Kiselev, Margarita Simonyan, Mikhail Novikov, Anton Selskii, Rodion Ukolov, Oksana Drapkina, Anna Orlova, Thomas Penzel, Anastasiya Runnova

**Affiliations:** 1 National Medical Research Center for Therapy and Preventive Medicine, Moscow, Russia; 2 Institute of Physics, Saratov State University, Saratov, Russia; 3 Institute of Cardiology Research, Saratov State Medical University, Saratov, Russia; 4 Interdisciplinary Sleep Medicine Center, Charité-Universitätsmedizin Berlin, Berlin, Germany

**Keywords:** Electroencephalography - EEG, Applied mathematics, Sleep disorders, Computational biophysics, Nonlinear phenomena

## Abstract

We performed a mathematical analysis of functional connectivity in electroencephalography (EEG) of patients with obstructive sleep apnea (OSA) (N = 10; age: 52.8 ± 13 years; median age: 49 years; male/female ratio: 7/3), compared with a group of apparently healthy participants (N = 15; age: 51.5 ± 29.5 years; median age: 42 years; male/female ratio: 8/7), based on the calculation of wavelet bicoherence from nighttime polysomnograms. Having observed the previously known phenomenon of interhemispheric synchronization deterioration, we demonstrated a compensatory increase in intrahemispheric connectivity, as well as a slight increase in the connectivity of the central and occipital areas for high-frequency EEG activity. Significant changes in functional connectivity were extremely stable in groups of apparently healthy participants and OSA patients, maintaining the overall pattern when comparing different recording nights and various sleep stages. The maximum variability of the connectivity was observed at fast oscillatory processes during REM sleep. The possibility of observing some changes in functional connectivity of brain activity in OSA patients in a state of passive wakefulness opens up prospects for further research. Developing the methods of hypnogram evaluation that are independent of functional connectivity may be useful for implementing a medical decision support system.

## Introduction

Obstructive sleep apnea (OSA) is characterized by recurrent occlusions of the upper airways, complete (apnea) or partial (hypopnea). Such occlusions result in intermittent hypoxemia, autonomic disturbances, and fragmentation of sleep with reduced deep sleep time and subsequent daytime sleepiness^1^. OSA is diagnosed if the apnea/hypopnea index (AHI) amounts to over five apneic/hypopneic episodes per hour of sleep^2^. Approximately 34% of men and 17% of women over 18 years of age meet the diagnostic criteria for OSA^3–5^. Despite high prevalence of OSA among patients with heart disease, sleep breathing disorders are habitually not diagnosed and treated in routine clinical practice due to apnea-induced stressors and adverse cardiovascular outcomes.

However, the impact of OSA on the human body is apparently systemic: in other words, it not only prevents the patient from obtaining enough sleep via fragmenting night sleep, but also disrupts feedback loops and interaction structures in various functional systems of the organism^6^. Hypoxemia, observed in patients with OSA, is a syndrome preventing sleep-related restorative processes and causing chemical or structural cellular damage to central nervous system, including injury due to an increase in the blood–brain barrier permeability^7^.

Currently, on the basis of imaging studies, it was established that the brain of patients with OSA is characterized by a decrease in gray matter volume, along with alterations in white matter integrity and activity at rest^8–11^. In addition, OSA patients exhibit a steady change in the power of neurophysiological correlates on their nocturnal and diurnal electroencephalograms (EEG) and during magnetoencephalography (MEG). Specifically, there are studies that credibly demonstrate spatial and frequency-related changes in sleep microstructure in patients with sleep apnea and simple snoring^12–14^. It was shown that daytime EEG in patients with OSA undergoes changes. For instance, a decrease in the ability to control attention in patients with sleep apnea correlated with the AHI value, which was accompanied by a change in the ratio of the powers of slow (delta/theta) and fast (alpha/beta) rhythms on the electroencephalogram^15,16^.

Various stages of sleep are also studied from the standpoint of identifying neural generators that are sources of specific oscillatory activity. For example, in^17–20^, the issues of identifying these sources and methods for their detection in EEG and MEG records of the brain activity, including during NREM sleep stages, were studied in great detail. Studies of REM sleep are often of a comparative nature, involving the issues regarding a person being awake before sleep, the nature of dreams^21^, and the fundamental heterogeneity of this sleep stage^22^. In addition to direct spectral analysis of brain activity, methods for frequency-time estimation of EEG envelope^23^ were offered.

Such analysis of the power of oscillatory components of superficially recorded brain activity can provide important information about simple stable EEG characteristics that could potentially play the role of diagnostic objective biomarkers of pathological processes. At the same time, such results are statistical in their nature, without providing information on stable EEG patterns, possibly foreshadowing the development of certain neurological consequences. In this regard, current methods for studying connectivity in multichannel EEG records seem more relevant. The EEG/MEG recording methods make it possible to characterize the activity of brain neurons that form time series in terms of functional connection^18,24,25^. Currently, the study of functional connections in brain activity can be based on various methods for assessing mutual information and transfer entropy^26^, correlation and coherence^27^, different types of synchronization^28–30^, etc. Basically, different functional connectivity assessment methods can give different results, because they are based on different underlying mathematical assumptions or measures of dependence^28^. However, similar results were shown on mathematical nonlinear models using linear correlation measures and coherence estimates based on Fourier and wavelet transforms^24^.

In this paper, we examine the structural properties of functional connectivity in the human brain during the nocturnal sleep based on the concepts of synchronization in chaotic systems^31,32^. We use the wavelet bicoherence (WB) to estimate the strength of interaction between the brain areas as measure of synchronization between EEG channels. The WB has proved itself as very powerful instrument to quantify the interactions of various biological systems^33–35^, including brain activity.

Currently, WB method for estimating and modeling functional connectivity of different EEG frequency bands is widely used in the study of cognitive processes, in particular, of attention function^29^. At the same time, there is evidence that functional connections are associated with chemical or structural cellular damage to the brain^36^. An analysis of the interaction of different brain activity areas in patients with OSA could predict developing neuronal disorders in pathological conditions of sleep disturbance, hypoxia, etc. Recent studies suggested that changes in the spatial characteristics of EEG functional connectivity were significant in all major neurophysiological frequency bands^37–39^. For instance, research on the connectivity of brain activity in OSA patients during nocturnal sleep^37,38^ demonstrated that the level of EEG interhemispheric synchronization declined vs. healthy volunteers. Most research in the field of functional connectivity of the brain during sleep has focused on the mathematical processing of only two EEG signals recorded in symmetrical channels. e.g., in early studies, polysomnography (PSG) recording included solely a couple of channels (C3, C4), which allowed considering the activity just of sensorimotor cortex projection.

The current limitations of processing and math modeling functional connectivity in brain activity are due to a number of factors. First, nocturnal sleep records have long duration, which complicates the processing of such big data sets. In particular, the use of familiar MATLAB modules and similar universal knowledge intensive systems for studying such volumes of data is difficult because of their insufficient adaptation to managing large amounts of numerical data. Besides, even the use of specific software adapted for parallel computing requires sufficiently powerful computer equipment. The solution to these computational processing problems is often achieved by using more simple methods for estimating the coupling strength only for the most pronounced frequency components of the oscillatory activity or by estimating linear correlation functions between biomedical signals. Second, it is customary to limit the processing of nighttime PSG records solely to EEG fragments according to certain stages of hypnograms. Despite the universal process of sleep staging in accordance with the American Academy of Sleep Medicine (AASM) criteria^40^, the resulting hypnograms of two experienced sleep experts may differ within 10–20%.

As a result, research into the functional connectivity of the brain during sleep has been largely restricted to limited calculations of linear correlation and phase synchronization characteristics between paired signals. Besides, numerical processing of only small selected fragments rather than the entire duration of the recording limits the results to expert hypnogram constructions. Consequently, this approach leaves out a significant amount of information about the dynamic interactions of various oscillatory regimes during the night. Apparently, these limitations of mathematical processing can lead to some contradictory results of EEG functional connectivity assessments: they can demonstrate the independence of connectivity from assessments of the state of the patient’s cognitive functions^41,42^, or exhibit the dependence pattern^43,44^. Therefore, the generalizability of many published studies on this subject is questionable.

In this paper, we are trying to approach the analysis of the functional connectivity in the patient brain during nocturnal sleep in a different way—viz., by considering the entire record of nocturnal sleep based on the records of six conventional EEG channels. Our study seeks to elucidate the structure of changes in brain functional connectivity caused by OSA syndrome. In addition, we put forward a hypothesis that the changes in the structure of EEG activity connections observed in OSA patients are very robust, as a result of which a deteriorated structure of connections can be detected when analyzing the entire sleep record without taking into account the hypnogram. This approach to the analysis of brain activity is promising for the development of independent assessment of disorders in OSA patients. Such assessment could provide additional information in evaluating the severity of the disease, which may be of interest from the standpoint of developing practical approaches to personalized medicine^45^.

Based on the contemporary WB method, we examined the dynamics of the interactions between various spatial areas of the brain in order to obtain additional data on significant changes in brain EEG connectivity in OSA. Our results expand our understanding of the fall in the magnitude of symmetrical interhemispheric connections and the growth of intrahemispheric connections in OSA patients through a detailed analysis of changes in the synchronization of ‘fast’ and ‘slow’ processes that are present in the frontal, central, and occipital EEG channels.

## Results

Our study presents the results of investigating the synchronization of brain activity in two groups of volunteers: control group (Group I) consisting of healthy participants of different ages and Group II including OSA patients. Standard high-quality PSG records were selected from the SIESTA database^46,47^. Test subjects were chosen to minimize the impact of comorbid diseases and complications related to physical and mental distress. The database included two PSG records for each participant, which made it possible to assess not only intraindividual, but also interindividual robustness in patterns of brain activity.

### Staging of nocturnal sleep in the first and second records

**Figure 1:**
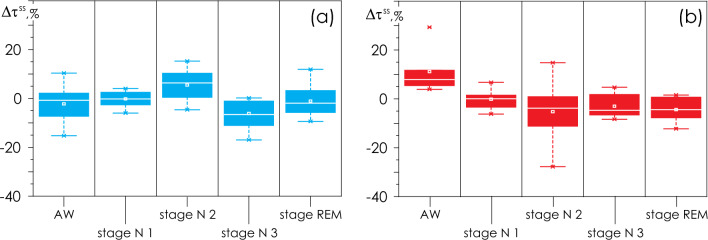
(**a**, **b**) $\Delta {\tau }^{SS}$—changes of relative time durations τ for each stage of PSG monitoring from the first to the second recording session of patients in Groups I and II, respectively. The following states are shown: AW—staying awake; sleep stages N1, N2, N3 and REM. Changes are shown as a percentage. The diagrams depict the following statistical characteristics of numerical indicators: the first and third quartiles (25–75%, inside the box); the median and the mean (transverse line and point inside the box, respectively); 1.5 interquartile ranges (shown by whiskers); and outliers represented by asterisks.

The assessment of $\Delta {\tau }^{SS}$ changes in the duration of sleep stages in patients revealed no statistically significant differences between groups I and II (Fig. 1). At the second monitoring session, Group I patients increased the duration of their N3 stage of deep sleep; whereas in patients of Group II, on average, the duration of all sleep stages slightly increased. Besides, Group II patients were waking up less frequently on the second overnight monitoring. Also, we observed the highest variability of N2 stage in patients with sleep apnea.

**Figure 2:**
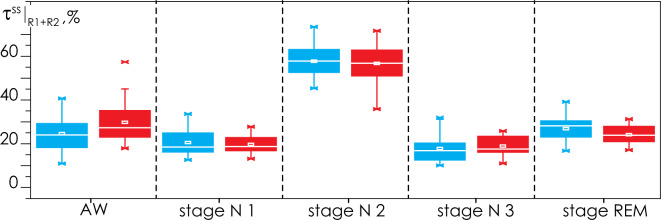
Statistical characteristics of the relative durations of each stage,${\left.\left\{{\tau }^{AW}; {\tau }^{N1}; {\tau }^{N2}; {\tau }^{N3}; {\tau }^{REM}\right\}\right|}_{R1+R2}$, defined for the first and second PSG records, averaged for Groups I (apparently healthy test subjects, in blue) and II (OSA patients, in red). Relative durations of stages are shown as a percentage of the total duration of the corresponding record. The diagrams depict the following statistical characteristics of numerical indicators: the first and third quartiles (25–75%, inside the box); the median and the mean (transverse line and point inside the box, respectively); 1.5 interquartile ranges (denoted by whiskers); and outliers represented by asterisks.

Figure 2 presents the statistical characteristics of the relative durations of each stage, $\left. {\left\{ {\tau^{AW} ;\;\tau^{N1} ;\;\tau^{N2} ;\;\tau^{N3} ;\;\tau^{REM} } \right\}} \right|_{R1 + R2}$, defined in the first and second PSG records. It is clearly seen that the duration of sleep stages and of nocturnal wakefulness for Groups I and II are quite homogeneous and do not differ statistically from each other. Overall, the sleep structure in patients of both groups is similar.

Based on comparable dynamics and statistically similar distributions of nocturnal stage durations during the first and second PSG monitoring sessions, we will further analyze the connectivity characteristics of brain activity without dividing them between the first and second nights.

### EEG connectivity strength distribution

**Figure 3:**
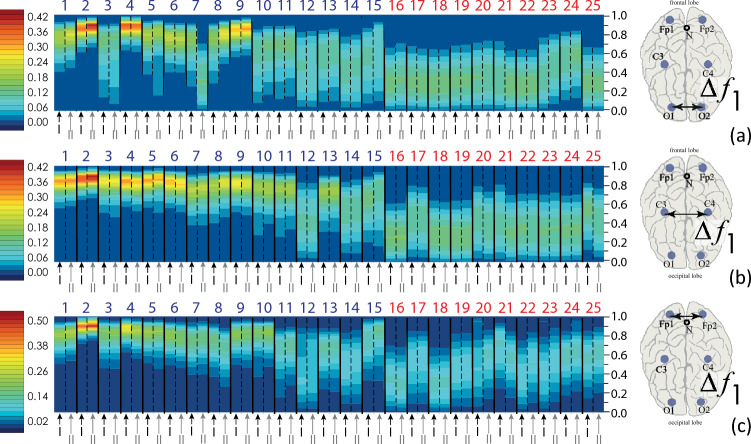
Distributions of the interhemispheric connectivity strength, $\rho \left({\mathrm{WB}}_{{\mathrm{EEG}}_{i},{\mathrm{EEG}}_{j}}^{{\Delta f}_{1}}\right),$ for the frequency band ${\Delta f}_{1}$: (**a**) EEG channels O1 and O2, (**b**) EEG channels C3 and C4, (**c**) EEG channels Fp1 and Fp2. The y-axis corresponds to the magnitude of the connection strength from zero to one. The surface color corresponds to the value of the frequency of occurrence of each of the values of the connection strength between the corresponding EEG channels, where the red color corresponds to the maximum value of the WB distribution density, while the dark blue color corresponds to the minimum value. For each pair of channels, a scale of connection strength values is given. At the bottom, alternating black and gray arrows I and II indicate the first and second PSG monitoring sessions for each patient. At the top of the graphs, the serial numbers of patients are shown. Group I patients are coded in blue, while Group II subjects are listed in red.

The distributions of interhemispheric connections, $\rho \left({\mathrm{WB}}_{{\mathrm{EEG}}_{i},{\mathrm{EEG}}_{j}}^{{\Delta f}_{k}}\right)$, are described by a complex structure of the connection strength dynamics during the first and second nights of monitoring sessions. Furthermore, they are described by the distribution range rather than only by the mean\median and maximum values. Figure 3 shows typical shapes of such distributions for the interhemispheric connectivity strength. The distributions $\rho \left({\mathrm{WB}}_{i,j}^{{\Delta f}_{1}}\right),$ lined up by the ordinal numbers of the subjects, demonstrate a high degree of robustness of the functional connectivity for the first and second PSG monitoring sessions of each participant. In addition, the average level of $\rho \left({\mathrm{WB}}_{i,j}^{{\Delta f}_{1}}\right)$ for the interhemispheric connections significantly increases in apparently healthy individuals against the background of patients with apnea (on the right of the diagrams in Fig. 3).

**Figure 4:**
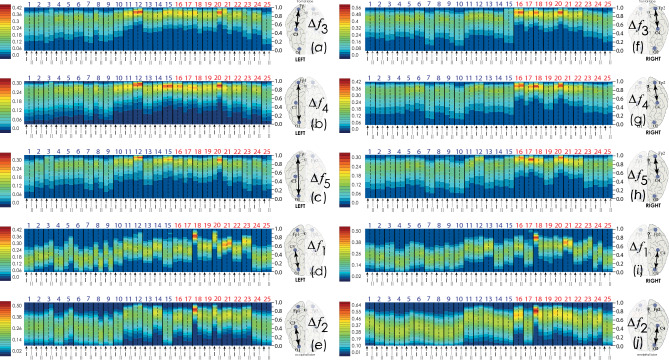
Distributions of the intrahemispheric connectivity strength, $\rho \left({\mathrm{WB}}_{{\mathrm{EEG}}_{i},{\mathrm{EEG}}_{j}}^{{\Delta f}_{k}}\right)$: (**a**) EEG channels Fp1 and C3, $\Delta {f}_{3}$; (**b**) EEG channels Fp1 and O1, $\Delta {f}_{4}$; (**c**) EEG channels Fp1 and O1, $\Delta {f}_{5}$; (**d**) EEG channels C3 and O1, $\Delta {f}_{1}$, (**e**) EEG channels C3 and O1, $\Delta {f}_{2}$; (**f**) EEG channels Fp2 and C4, $\Delta {f}_{3}$; (**g**) EEG channels Fp2 and C4, $\Delta {f}_{4}$; (**h**) EEG channels Fp2 and C4, $\Delta {f}_{5}$; **(i)** EEG channels C4 and O2, $\Delta {f}_{1}$; (**j**) EEG channels C4 and O2, $\Delta {f}_{2}$. The y-axis corresponds to the magnitude of the connection strength from zero to one. The surface color corresponds to the value of the frequency of occurrence of each of the values of the connection strength between the corresponding EEG channels, where the red color corresponds to the maximum value of the WB distribution density, while the dark blue color corresponds to the minimum value. For each pair of channels, a scale of connection strength values is given. At the bottom, alternating black and gray arrows I and II indicate the first and second PSG monitoring sessions for each patient. At the top of the graphs, the serial numbers of patients are shown. Group I patients are coded in blue, while Group II subjects are listed in red.

Similar distributions of the connectivity strength, $\rho \left({\mathrm{WB}}_{{\mathrm{EEG}}_{i},{\mathrm{EEG}}_{j}}^{{\Delta f}_{k}}\right)$, for various intrahemispheric pairs of EEG channels are shown in Fig. 4. The structure of the distributions seems stationary. The maximum level of connection strength and the distribution range for nearly every patient are repeated on the first and second nights of sleep. Moreover, the general shape of probability distributions is robust for all pairs of channels in the considered frequency bands.

At the same time, in patients Nos. 18 and 20, the results of the synchronization assessment between the occipital channels, show significant differences for some frequency bands, when comparing the first and second PSG records, as seen in Fig. 4d, e, i, j. This situation is typical in the analysis of biomedical signals generated by living systems; it could be caused by as technical issues regarding the recording procedure on the first and second nights of a study subject sleep, as certain individual circumstances of patients.

The graphical representation of distributions of the connectivity strength, $\rho \left({\mathrm{WB}}_{{\mathrm{EEG}}_{i},{\mathrm{EEG}}_{j}}^{{\Delta f}_{k}}\right)$, is cumbersome and redundant. Besides, examination of the synchronization distribution does not allow a direct statistical assessment of the differences in the characteristics of the first and second nocturnal sleep sessions between Groups I (virtually healthy participants)) and II (OSA patients).

### Changes of EEG connectivity strength between the first and second records

To assess the robustness of the EEG connectivity characteristics, we calculated the mean values of connectivity strength, $\left\langle {{\text{WB}}_{{{\text{EEG}}_{i} ,{\text{EEG}}_{j} }}^{{\Delta f_{k} }} } \right\rangle_{1,2}$, over the entire duration of the first and second PSG sessions.

**Figure 5:**
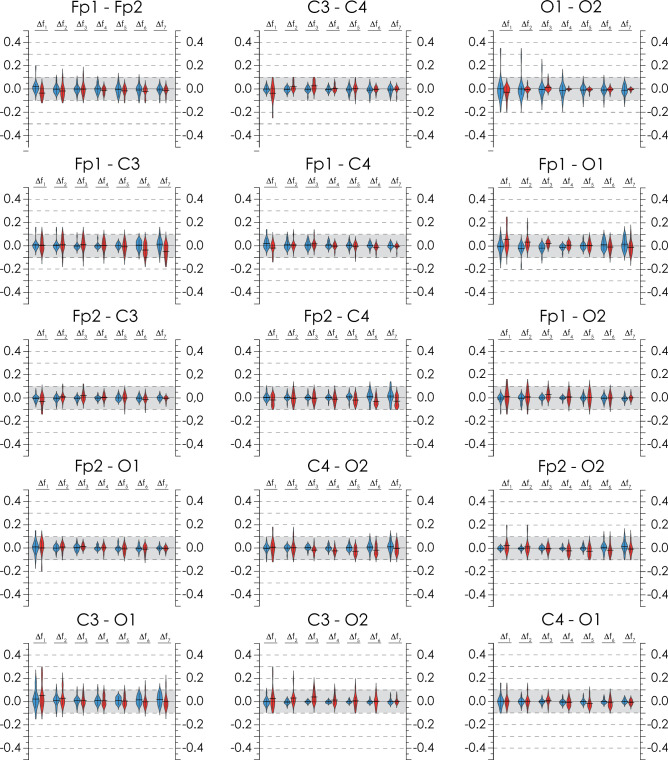
Diagrams of differences in synchronization levels for the frequency bands ∆*f*_1_–∆*f*_7_ assessed during the first and second nights of PSG records. Each set of diagrams is presented for a specific pair of EEG channels, the standard notations of which are indicated on the top. The gray background shows the range of differences in synchronization estimates for the first and second nights within [−0.1; 0.1]. Diagrams in blue and red represent calculation results for Groups I and II, correspondingly.

The results of statistical analysis of the connectivity change in EEG records, $\left. {\Delta \left( {\left\langle {{\text{WB}}_{{{\text{EEG}}_{i} ,{\text{EEG}}_{j} }}^{{\Delta f_{k} }} } \right\rangle } \right)} \right|_{1 - 2}$, are shown in Fig. 5, the diagrams on which demonstrate the following general trends. First, the differences in the average synchronization from night to night tend to 0 without exceeding 0.1 in absolute value (i.e., they are minimal). Second, the distributions of the calculated values are close to normal, and their mean also tends to zero. Finally, outliers up to 0.2–0.3 in some frequency bands occur in the records of only one or two patients and can be due to either typical errors in the registration of biomedical signals or high individual variability within the population.

The performed estimates imply a high stationarity, stability and steadiness in the oscillational structure of brain activity. This structure remains almost unchanged when analyzing two independent records of nocturnal sleep. Thus, it seems possible to pool together two PSG records in order to increase the reliability of the results.

### Changes of EEG connectivity strength at different stages of polysomnography

Next, we assessed the structural robustness of electroencephalographic connectivity in brain activity directly during PSG, i.e., taking into account the resulting hypnogram and structuring the entire duration of sleep into standard stages of NREM sleep (N1, N2, N3), REM sleep, and arousal wakefulness.

**Figure 6:**
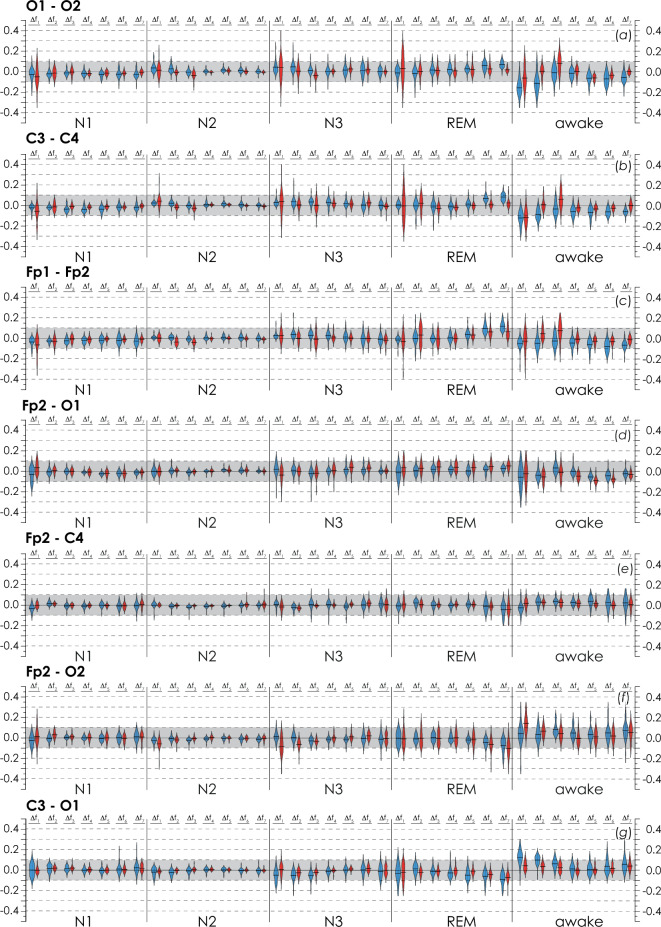
Distribution diagrams for $\left. {\Delta \left( {\left\langle {{\text{WB}}_{{{\text{EEG}}_{i} ,{\text{EEG}}_{j} }}^{{\Delta f_{k} }} } \right\rangle } \right)} \right|_{{{\text{stage}} - 1,2}}$ calculated for stages of NREM sleep (N1, N2, N3), REM sleep, and arousal wakefulness. For each set of diagrams, a pair of channels for which the calculation was performed is indicated. The gray background depicts the range of differences in synchronization estimates for the first and second nights within [−0.1; 0.1]. The calculation results for Groups I and II are coded in blue and red, respectively.

For each pair of EEG channels, recorded in each study participant, we measured a changes in the connectivity strength at a certain PSG stage, relative to the average overnight connectivity strength, $\left\langle {{\text{WB}}_{{{\text{EEG}}_{i} ,{\text{EEG}}_{j} }}^{{\Delta f_{k} }} } \right\rangle_{{1,2}}$. Figure 6 presents statistical estimates of differences, $\left. {\Delta \left( {\left\langle {{\text{WB}}_{{{\text{EEG}}_{i} ,{\text{EEG}}_{j} }}^{{\Delta f_{k} }} } \right\rangle } \right)} \right|_{{{\text{stage}} - 1,2}}$, calculated for some pairs of EEG channels at each stage of a hypnogram for patients in Groups I and II. Similar difference diagrams for the remaining pairs of channels are presented in Appendix I.

First, the analysis of the presented diagrams allowed observing that the range of values of $\left. {\Delta \left( {\left\langle {{\text{WB}}_{{{\text{EEG}}_{i} ,{\text{EEG}}_{j} }}^{{\Delta f_{k} }} } \right\rangle } \right)} \right|_{{{\text{N}}1,{\text{N}}2, {\text{N}}3 - 1,2}}$ for stages N1–N3 of NREM sleep was minimal, and their means belonged to the interval of [−0.05; 0.05]. Outliers of variability in N3 deep sleep were associated with a random increase in the level of variability in one study participant, while the shape of the distribution was indicative of its normality.

Then, the stage of REM sleep slightly increased the variability of the functional connectivity in electroencephalographic activity. The increase in the value of $\left. {\Delta \left( {\left\langle {{\text{WB}}_{{{\text{EEG}}_{i} ,{\text{EEG}}_{j} }}^{{\Delta f_{k} }} } \right\rangle } \right)} \right|_{{{\text{REM}} - 1,2}}$ was more pronounced for interhemispheric connections, as seen in Fig. 6a–c. In low frequency bands, $\left. {\Delta \left( {\left\langle {{\text{WB}}_{{{\text{EEG}}_{i} ,{\text{EEG}}_{j} }}^{{\Delta f_{k} }} } \right\rangle } \right)} \right|_{{{\text{REM}} - 1,2}}$ prevailed in OSA patients (Group II), while in high frequency bands, the effect was more noticeable in apparently healthy study participants (Group I). However, the overall variability remained low. The average values of $\left. {\Delta \left( {\left\langle {{\text{WB}}_{{{\text{EEG}}_{i} ,{\text{EEG}}_{j} }}^{{\Delta f_{k} }} } \right\rangle } \right)} \right|_{{{\text{REM}} - 1,2}}$ did not go beyond the interval of [−0.1; 0.1].

Finally, the maximum variability was associated with arousal wakefulness. Of course, this aspect of the results was not unexpected, since the processes of enhancing participants’ response to the environment, along with activation of cognitive functions and self-awareness per se, increased individual variability in the EEG functional structure.

At the same time, the main changes in stage of arousal wakefulness were observed only in the low-frequency bands of delta and theta oscillations. For all considered pairs of EEG channels, we observed only slight changes of the distributions and means in the range of [−0.2; 0.2], while outliers did not exceed 0.35–0.39. So, a very interesting and surprising phenomenon was the EEG functional connectivity robustness during awakenings.

The performed analysis of changes in the EEG connectivity strength between the stages of hypnograms allows proposing that the main structure of functional connectivity in study participants at different stages of sleep remains very similar. The analysis of changes in the strength of EEG connectivity between the stages of hypnograms allows us to observe that the main structure of functional connectivity in the participants of the study at different stages of sleep remains practically unchanged. Deviations of connection strengths in each sleep stage relative to the nightly average connection strength are insignificant, mainly being in the range [−0.1; 1.0]. In other words, at any stage of sleep, deviations in the measure of synchronization, calculated from the average measure for the night, usually do not exceed 10%. Therefore, averaging the measure of synchronization over the entire duration of nocturnal sleep is not the extreme oversimplification of the situation and may be a quite adequate method for estimating the strength of connectivity between pairs of EEG channels. Consequently, the approach of pairwise estimates of EEG connectivity strength calculated over the entire duration of nocturnal sleep does not contradict results, described in this Section.

### Statistical estimates of EEG connectivity strength

**Figure 7:**
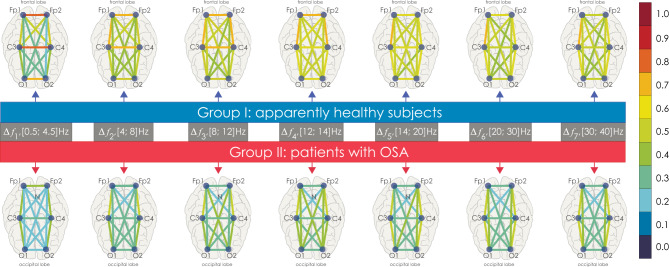
Graphical diagrams of mean connectivity strength values between various EEG channels. The connectivity strength is color-coded according to the legend on the right. The frequency bands ∆*f*_1_–∆*f*_7_ are labeled in the middle of the figure against a gray background. The connectivity patterns in the groups of healthy participants and OSA patients are shown above and below, respectively.

The calculated $\left\langle {{\text{WB}}_{{{\text{EEG}}_{i} , {\text{EEG}}_{j} }}^{{\Delta f_{k} }} } \right\rangle$ connectivity strength means for groups I and II for all pairs of EEG channels are presented in Fig. 7. EEG activity demonstrates maximum connectivity in interhemispheric symmetrical connections (Fp1–Fp2, C3–C4, O1–O2) for the slowest oscillations (∆*f*_1_). In general, the average strength of associations in OSA patients varies more or less in all frequency bands, compared with the control group.

**Figure 8:**
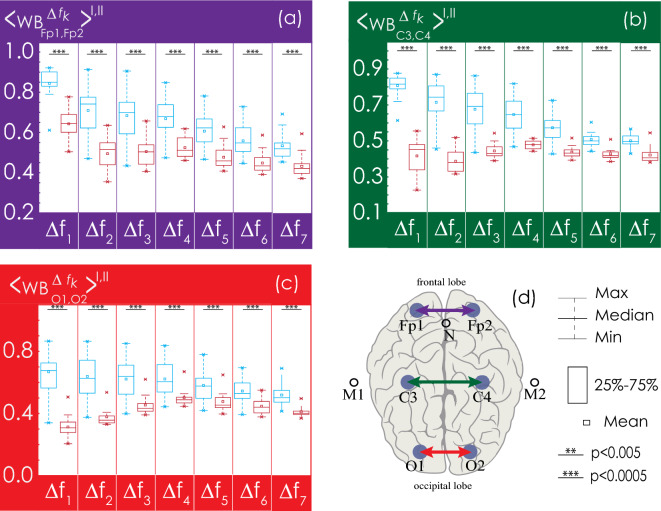
(**a**–**c**) Distributions of connectivity strength, $\left\langle {{\text{WB}}_{i,j}^{{\Delta f_{k} }} } \right\rangle^{{{\text{I}},{\text{ II}}}}$, in Groups I (blue) and II (red) for interhemispheric connections: among frontal leads (Fp1 and Fp2), central leads (C3 and C4), and occipital leads (O1 and O2), respectively. (**d**) Arrangement of EEG channels and interhemispheric connections. Interpretation of the symbols used in diagrams.

**Figure 9:**
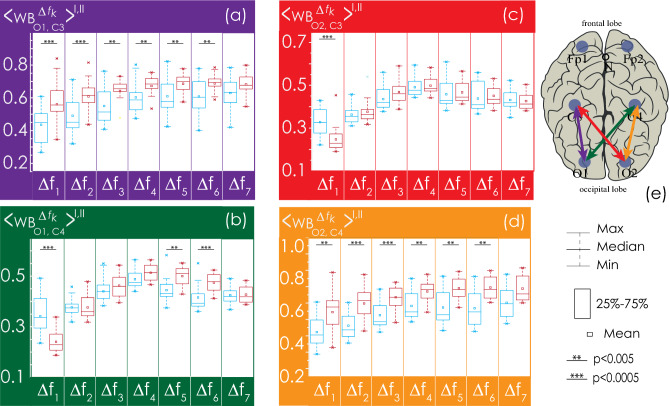
(**a**–**d**) Distributions of connectivity strength, $\left\langle {{\text{WB}}_{i,j}^{{\Delta f_{k} }} } \right\rangle^{{{\text{I}},{\text{ II}}}}$, in Groups I (blue) and II (red) for interactions between EEG channels (O1 and C3, O1 and C4, O2 and C3, O2 and C4, respectively). (**e**) Arrangement of EEG channels and interhemispheric connections. Interpretation of the symbols used in diagrams.

**Figure 10:**
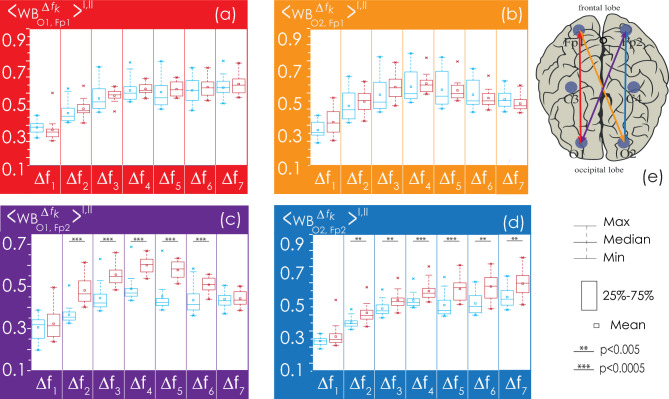
(**a**–**d**) Distributions of connectivity strength, $\left\langle {{\text{WB}}_{i,j}^{{\Delta f_{k} }} } \right\rangle^{{{\text{I}},{\text{ II}}}}$, in Groups I (blue) and II (red) for interactions between EEG channels O1 and Fp1, O1 and Fp2, O2 and Fp1, O2 and Fp2, respectively. (**e**) Arrangement of EEG channels and interhemispheric connections. Interpretation of the symbols used in diagrams.

**Figure 11:**
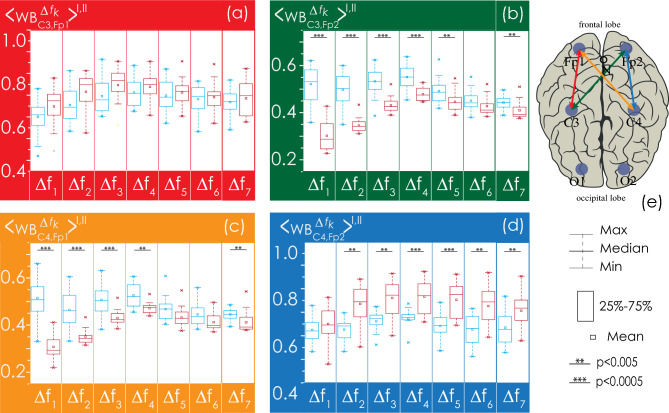
(**a**–**d**) Distributions of connectivity strength, $\left\langle {{\text{WB}}_{i,j}^{{\Delta f_{k} }} } \right\rangle^{{{\text{I}},{\text{ II}}}}$, in Groups I (blue) and II (red) for interactions between EEG channels C3 and Fp1, C3 and Fp2, C4 and Fp1, C4 and Fp2, respectively. (**e**) Arrangement of EEG channels and interhemispheric connections. Interpretation of the symbols used in diagrams.

Next, we consider in detail the statistical characteristics of changes in EEG connectivity for the Groups I and II. The consideration begins with the analysis of interhemispheric connectivity presented in Fig. 8. The strength of bilateral connections between the symmetrical channels (O1–O2, C3–C4, Fp1–Fp2) in healthy patients (Group I, shown in blue in Figs. 8, 9, 10, 11) is significantly higher than in patients with apnea (Group II, shown in red in Figs. 8, 9, 10, 11). However, the group of apparently healthy patients exhibits significant individual differences in the strength of the connectivity, and the characteristics of bilateral connections in patients with apnea are surprisingly homogeneous (i. e., they are characterized by a very small variation range), a detailed numerical analysis of which is presented in Appendix II. The values of the observed bilateral connections between Groups I and II are the highest for oscillatory processes in frequency bands ${\Delta f}_{1-5}$. They become less pronounced for the fastest processes (${\Delta f}_{6})$.

In Fig. 9, the interaction of processes between the left occipital channel (O1) and the central channels (C3 and C4) could also be described as significantly different between the studied groups. Within one hemisphere, the strength of connectivity in the presence of apnea syndrome increases significantly, compared with apparently healthy subjects. When considering interhemispheric interaction, i.e., between channels O1 and C4, the slowest oscillatory processes in the frequency band ${\Delta f}_{1}$ are more synchronized in apparently healthy participants of Group I, and the synchronization degree of fast oscillations (${\Delta f}_{5-6}$) is higher in patients with apnea. The pattern of symmetrical interactions between the right occipital channel (O2) and the central channels is less diverse. In case of interhemispheric interactions (O2–C3) in the band ${\Delta f}_{1}$ in patients with apnea (Group II), the degree of synchronization decreases; however, there are no differences in the connectivity strength for higher frequencies. At the same time, within the right hemisphere, the strength of interaction (O2–C4) in Group II significantly exceeds that in Group I for all frequency bands.

As seen in Fig. 10, for the farthest distances, corresponding to the connections between the left/right occipital channels (O1/O2) and the frontal channels (Fp1/Fp2), the strength of connectivity increases with the speed of the oscillatory processes, reaching maximum values in band ${\Delta f}_{4}$. When considering the interaction of oscillatory processes in both occipital channels and the left frontal channel (Fp1), the results of the analysis of the groups do not differ statistically. For the EEG of the right frontal lead (Fp2), the values of connectivity with the signals of the occipital channels for Group II significantly exceed those for Group I in bands ${\Delta f}_{2-6}$, differing to the maximum in bands ${\Delta f}_{4-5}$.

As seen in Fig. 11, in the left hemisphere, the strengths of interaction between the oscillatory processes of the frontal lead (Fp1) and the central channel (C3) do not differ between the study participants of two groups. At the same time, when analyzing symmetrical activity in the right hemisphere (Fp2 and C4), observed in Group II (patients with apnea), the strength of interaction significantly exceeds the observed connectivity in apparently healthy participants for oscillatory processes in bands ${\Delta f}_{2-5}$. When analyzing the synchronization of interhemispheric EEG activity recorded in Fp1 and C4, as well as in Fp2 and C3, the strength of the connectivity between slow oscillatory processes in frequency bands ${\Delta f}_{1-4}$ decreases in Group II vs. Group I.

## Discussion

As a detailed analysis of the frequency synchronization pattern demonstrates, the presence of OSA leads to a significant restructuring of the functional connections in the brain neuronal activity recorded on the EEG. Considering the large volume of our study, we briefly summarize the results of the identified patterns. First of all, we demonstrated a significant robustness of the functional connectivity structure in patients both among different nocturnal sleep monitoring sessions and between different stages of nocturnal sleep. We paid special attention to changes in the structure of brain functional connectivity observed during REM sleep. Castro et al*.*^48^ showed in their experimental animal studies that the coherence of gamma activity, when analyzed in terms of intrahemispheric and interhemispheric connections, was reduced during REM sleep vs. other behavioral states. In our study, we observed changes in the synchronization level for the REM sleep stage in the high-frequency band as well. However, surficial registration of brain activity in humans does not allow analyzing high frequencies (up to 100 Hz). Overall, intrahemispheric connections demonstrated a decrease in the coherence of EEG signals, while interhemispheric connections, on the contrary, increased to some extent. Furthermore, such pattern was observed both in the group of healthy participants and in OSA patients.

**Figure 12:**
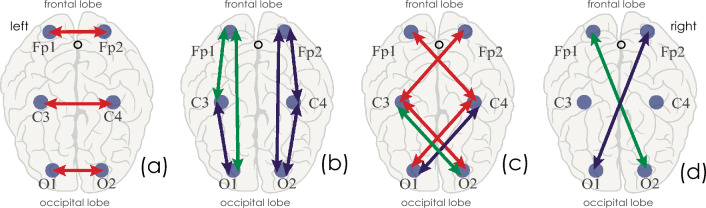
Scheme of identified patterns in connectivity changes in Group II (OSA patients) vs. control group (Group I). Connections for which the synchronization has decreased are depicted in red. Connections with an increased synchronization are shown in blue. Connections with unchanged synchronization are marked in green. (**a**) Change in symmetrical interhemispheric connections; (**b**) change in intrahemispheric connections; (**c**) asymmetric interhemispheric short-distance connections; (**d**) asymmetric interhemispheric long-distance connections. Coupled connections in panel (**c**) between channels C3–O2 and C4–O1 correspond to two patterns of dynamics: a reduction in the connectivity strength in the frequency band ${\Delta f}_{1}$ and stable/increased connectivity in the bands ${\Delta f}_{2-7}$ respectively.

In apnea, the connectivity dropped in symmetrical hemispheric leads for all frequency bands, as shown schematically in Fig. 12a. These results refine previously obtained results^39,49,50^ for symmetrical bilateral interhemispheric connections in patients with apnea. Sleep apnea patients exhibit a very homogeneous pattern of asynchronous dynamics for the occipital channels (O1 and O2), which may indicate the most significant disruption of this connection type between them.

Previously, a strong interaction between the respiration and infra-slow oscillations of the scalp EEG was established^51^. The maximum values of the coherence function between infra-slow EEG oscillations and respiration were observed in the occipital region (channels O1 and O2). Studies of spontaneous and stimulated breathing detected several central pattern generators (CPGs), based on self-sustained oscillator, in brain structures, with a basic frequency close to 0.1 Hz. These CPGs are responsible for generating infra-slow rhythms of cortical activity associated with rhythms of autonomic control of heart rate (with a base frequency of about 0.1 Hz) and blood pressure^52^, and exhibit similar synchronization with respiration^53^. However, an increase in the low-frequency synchronization of respiration and EEG, recorded in the occipital channels, may also be associated with the leakage of powerful stem activity, including the respiratory center, into the surface EEG signals. Nevertheless, despite the complexity and ambiguity of the findings on this issue, the presence of distinct changes in functional connectivity in the occipital lobe due to OSA requires further attention. To clarify these facts, additional experimental and theoretical work is required with invasive recording of signals from the cortical and nuclear structures of the laboratory animal brain under the control of their respiratory activity.

The synchronization in some leads within the hemispheres of OSA patients was increased vs. the control group, as schematically demonstrated in Fig. 12b with blue arrows. At the same time, some intrahemispheric connections in the left hemisphere remained unchanged (Fig. 12b, green arrows). Different dynamics in the hemispheres and their lobes is in good agreement with the recently discovered fundamentally different functional activity of the right and left hemispheres in people^54–56^. In particular, the recent publication by Malik-Moraleda et al.^57^, using fNIRS and fMRI, convincingly suggested that the implementation of the unique mechanism of human speech activates various areas of the left hemisphere, specifically, in the frontal lobe. In our study, the functional connectivity of the left hemisphere was indistinguishable between OSA patients and healthy study participants. Hence, we assume that the functional activity accompanied by speech processes is so complex and imperative for the personality structure that even with the powerful influence of the OSA syndrome, leading to the episodes of oxygen starvation, this structure cannot be disturbed.

In addition, the study of asymmetric interhemispheric connections demonstrated their very complex structure. The connections between the motor and frontal areas repeat the dynamics of symmetrical connections: they significantly decrease in Group II, compared with Group I, as seen in Fig. 12c. The analysis of channel connections in the occipital and central lobes demonstrated that the degree of synchronization in Group II (OSA patients) decreased in the course of analyzing the ‘slow’ activity in the ${\Delta f}_{1}$ frequency band and did not change (C3–O2) or even increased (C4–O1) in frequency bands ${\Delta f}_{2-7}$ (Fig. 12c, coupled arrows).

At long distances, interhemispheric connectivity strength between frontal and occipital areas either increases (Fp1–O2) or does not change (Fp2–O1), as demonstrated in Fig. 12d. It is possible that the reduction in the symmetric connectivity between the left and right hemispheres is compensated by an increase in synchronization within the right hemisphere and by an increase in the connectivity between the right occipital lobe and left central/frontal lobes.

We suggest that our hypothesis about the persistence of disturbances in the structure of brain activity in obstructive sleep apnea syndrome is rather plausible. In such case, a change in the functional connectivity due to OSA syndrome, recorded in EEG, can be considered from the standpoint of a mathematical model of the dynamic hysteresis process^58,59^. Hence, we can assume that the neurophysiological activity of the brain has two types of robust functional connectivity, I and II, observed in Groups I and II, correspondingly.

The weak impact of pathophysiological factors, such as hypoxia, increased blood pressure, and other factors accompanying OSA syndrome, leads to the transition of a robust state I of the brain activity dynamics to a robust state II. The restoration of a conditionally normal state upon removal of airway obstruction does not immediately lead to the return of the system to state I, which is observed on the example of of nocturnal awakenings in OSA patients.

Based on the regularities in the stationary functional activity structure in the left hemisphere EEG and the maximum power of changes in the projection area of the occipital cortex observed in patients with apnea, it can be assumed that some of these significant transformations of synchronization levels will be detectable in patients in a state of passive wakefulness^49,60^. In addition, this assumption correlates with changes in the power of high-frequency activity observed in OSA patients, e.g., in Grenèche et al*.*^15,16^. Moreover, it can be assumed that patients with OSA syndrome may exhibit significant changes in the existing robust networks of interhemispheric connections upon activation of cognitive functions, especially various types of attention.

Connections between the occipital lobes of the brain may be especially promising for the search for such diagnostic criteria of nocturnal sleep disorders.

## Materials and methods

### Experimental data

The study was conducted on depersonalized patient data from the SIESTA database^46,47^. Among other objectives, the SIESTA project (1997–2000, funded by the EU Commission under its 4th research framework), aimed to investigate the architecture of nocturnal sleep based on PSG, primarily EEG. Within the framework of the SIESTA project, PSG recordings were conducted on 194 apparently healthy subjects and 98 patients with sleep disorders, in particular, OSA syndrome. Our study included medical records of 25 patients distributed among two groups. The exclusion criteria were: severe disability, obesity, chronic diseases of the endocrine system (diabetes, hypo/hyperthyroidism), cardiovascular diseases (including arterial hypertension, coronary artery disease, chronic heart failure, etc.), neurological disorders (epilepsy, demyelinating diseases, etc.), neuropsychiatric pathologies (clinical depression, obsessive compulsive disorder, anxiety disorder), and impossibility of independent signing formal consent to participate in the study, including language-related difficulties.

Group I included apparently healthy study participants (N_1_ = 15; age: 51.5 ± 29.5 years; median age: 42 years; male/female ratio = 8/7). Group II encompassed subjects with OSA syndrome (N_2_ = 10; age: 52.8 ± 13 years; median age 49 years; male/female ratio = 7/3). Each patient participated in a PSG study twice (1–3 nights apart) in a specially equipped sleep laboratory. The duration of sleep was 7–9 h, from 21.30 to 23.00 to the patient’s usual awakening time. During the first- and second-night recordings, as well as between them, CPAP therapy was not carried out.

**Figure 13:**
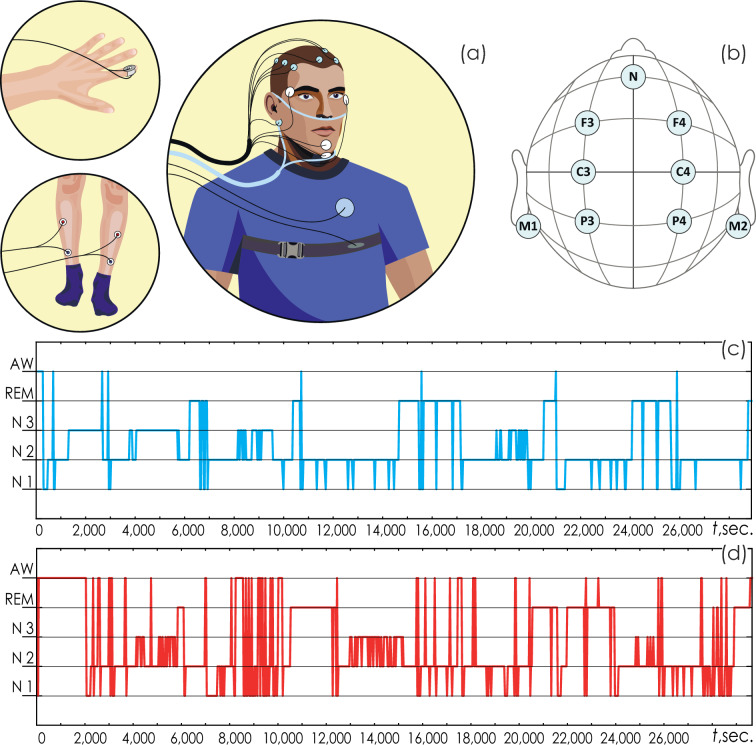
(**a**) The location of the main sensors recording various biomedical signals during polysomnography; (**b**) layout of EEG surface electrodes; (**c, d**) labeling of sleep stages (hypnogram) of the second night records in patients 12 and 23, respectively. The illustration was made using the Illustrator CS3 (License Certificate under the Transactional License Program-Education for Saratov State University, ID CEO801213).

PSG was recorded during nocturnal sleep and included various signals: of the electrocardiogram (ECG), respiratory function, oculography (OCG), electromyogram (EMG), and electroencephalogram (EEG). The ECG signal was recorded in standard lead I according to Einthoven. Respiratory signals were recorded using an oronasal flow temperature sensor and a snore sensor. EMG signals were recorded on the patient’s right forearm and left calf. OCG signals included records of horizontal and vertical eye movements. The locations of the main sensors in PSG are shown in Fig. 13a. EEG signals were recorded in 6 standard leads according to 10–20 scheme, as shown in Fig. 13b. EEG, ECG and respiratory function signals were filtered with the bandpass of 0.5–40 Hz and sampled at 100 Hz. All PSG records were reviewed by a certified sleep medicine physician for the purpose of nighttime sleep staging. Figures 13 (*c*) and (*d*) depict typical polysomnograms with stages N1–N3 of deep sleep, REM sleep stage, and episodes of nocturnal awakenings of two patients.

Tables containing the results of physical examination of study participants, as well as the clinical assessments of the PSG study, are included in Appendix III.

### Ethical statement

All procedures, performed in studies involving human participants, were in compliance with 1964 Declaration of Helsinki and its later amendments. All experimental data were approved by the Ethics Committee of Klinikum der Philipps-Universität Marburg, Germany. All study participants were over 18 years of age. They provided written informed consent to participate in the study.

### Analysis of differences between hypnograms of the first and second records

**Figure 14:**
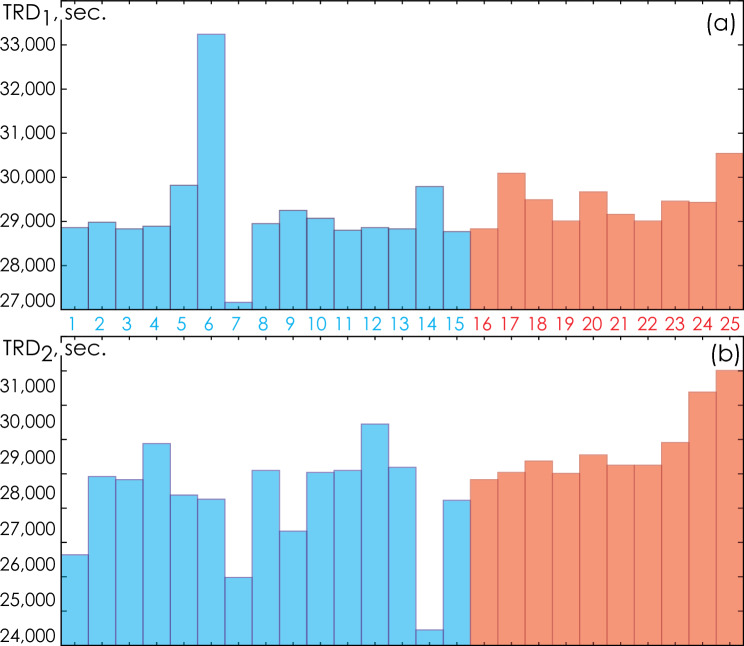
(**a**, **b**). Total durations of the first and second recording sessions, correspondingly. Group I participants are shown in blue, OSA patients (Group II) are marked in red.

For each record, the total record duration (TRD, sec) was estimated from the onset of sleep to the full awakening of the patient. Figure 14 presents the TRD values for the first and second monitoring sessions for each study participant.

Further, for each of the diagnosed stages of the first and second sleep records, the relative duration times τ were assessed as:

1 $$\tau_{R1}^{SS} = \mathop \sum \nolimits_{I} T_{R1}^{ssI} /TRD_{1} \cdot 100\% ;\quad \tau_{R2}^{SS} = \mathop \sum \nolimits_{I} T_{R2}^{ssI} /TRD_{2} \cdot 100\% ,$$

where SS is the symbol for each sleep stage on the hypnogram, specifically for arousal wakefulness (AW), NREM stages (N1, N2, N3), and REM stage; R1,2 stands for the first and second sleep records; ${T}_{R\mathrm{1,2}}^{ssI}$ is duration (in seconds) of each episode of sleep stage during the first and second recording sessions; and TRD_1,2_ is a total duration of the first and second sleep records combined.

The change in the relative durations τ of each sleep stage for the first and second patient records was estimated as follows:

2 $$\Delta \tau^{SS} = \left( {\tau_{R1}^{SS} - \tau_{R2}^{SS} } \right) \cdot 100\% .$$

### Analysis of multiscale connectivity in EEG

We used the WB to estimate the functional connectivity strength between the brain lobes. The WB is considered a very powerful tool for the quantification of the interactions between biomedical signals on various oscillatory scales^35,61,62^, including brain activity^29,62,63^. A detailed algorithm for calculating WB is presented in Appendix IV.

With a single WB, ${WB}_{{EEG}_{i},{EEG}_{j}}\left(f,t\right)$ = 1, the signals ${EEG}_{i}\left(t\right)$ and ${EEG}_{j}\left(t\right)$ are fully synchronous at time *t* at frequency *f*. Conversely, in the case of zero bicoherence, ${WB}_{{EEG}_{i},{EEG}_{j}}\left(f,t\right)$ = 0, the signals exhibit a fully asynchronous mode. Accordingly, ${WB}_{{EEG}_{i},{EEG}_{j}}\left(f,t\right)$, changing within the given boundary values [0; 1], provides complete information about the connectivity of signals on the time–frequency plane (*f*; *t*).

We considered integral bicoherence, calculated from pairs of EEG signals in seven frequency bands, ∆*f*_k_ [${f}_{1}^{k}; {f}_{2}^{k}$], Hz, as:

3 $$WB_{{EEG_{i} ,EEG_{j} }}^{{\Delta f_{k} }} \left( t \right) = \frac{1}{{\Delta f_{k} }}\mathop \smallint \limits_{{f_{1}^{k} }}^{{f_{2}^{k} }} WB_{{EEG_{i} ,EEG_{j} }} \left( {f,t} \right) \cdot df,$$

where *k* = 1… 7 is a number of the considered frequency band (Table 1).

**Table 1:** Characteristics of frequency bands for assessing the integral bicoherence of signals.

Frequency band (FB)	Lower limit of the FB,${f}_{1}^{k}$, Hz	Upper limit of the B,${f}_{2}^{k}$, Hz	FB width, *∆f*_*k*_* ,* Hz
*∆f*_1_	0.5	4.5	4
*∆f*_2_	4	8	4
*∆f*_3_	8	12	4
*∆f*_4_	12	14	2
*∆f5*	14	20	6
*∆f*_6_	20	30	10
*∆f*_7_	30	40	10

**Figure 15:**
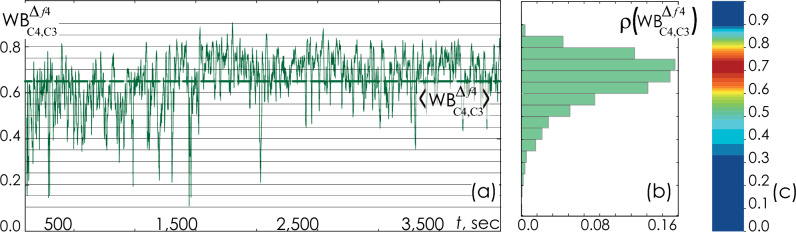
(**a**) A fragment of the time dependence of the integral bicoherence ${\mathrm{WB}}_{C4, C3}^{{\Delta f}_{4}}\left(t\right)$ (6), calculated from the signals recorded in the EEG channels C_3_ and C_4_ in the volunteer B0011, in the frequency band ∆*f*_*4*_ [12; 14] Hz. The horizontal dotted line shows the arithmetic mean of the wavelet bicoherence $\left\langle {{\text{WB}}_{C4, C3}^{{\Delta f_{4} }} } \right\rangle$. (**b**) Corresponding probability density distribution $,\rho \left({\mathrm{WB}}_{C4, C3}^{{\Delta f}_{4}}\right)$, calculated from the given fragment of the signal. (**c**) Surface representation of the probability density value $\rho \left({\mathrm{WB}}_{C4, C3}^{{\Delta f}_{4}}\right)$, where the red color corresponds to the maximum value of the bicoherence distribution density (~ 0.16), while the dark blue color corresponds to the minimum value (~ 0.0).

In this case, for each pair of signals, ${\mathrm{EEG}}_{i}\left(t\right)$ и ${\mathrm{EEG}}_{j}\left(t\right)$, seven options of time dependences, ${\mathrm{WB}}_{{\mathrm{EEG}}_{i},{\mathrm{EEG}}_{j}}^{{\Delta f}_{k}}\left(t\right),$ can be plotted. An example of such dependence is shown in Fig. 15a. For each patient, over the entire duration of the night recording, we evaluated the mean bicoherence $\left\langle {{\text{WB}}_{{{\text{EEG}}_{i} ,{\text{EEG}}_{j} }}^{{\Delta f_{k} }} } \right\rangle$ and standard deviation ${\Delta \mathrm{WB}}_{i,j}^{{\Delta f}_{k}}$ for pairs of EEG channels. In addition, the probability distribution of the WB strength, $\rho \left({\mathrm{WB}}_{{\mathrm{EEG}}_{i},{\mathrm{EEG}}_{j}}^{{\Delta f}_{k}}\right)$, was constructed, as shown in Fig. 15b. The value of $\rho \left({\mathrm{WB}}_{{\mathrm{EEG}}_{i},{\mathrm{EEG}}_{j}}^{{\Delta f}_{k}}\right)$ is normalized in such a way that the area occupied by the geometric figure depicting the probability density distribution is equal to 1.

Shown in Fig. 15c graphical interpretation of the probability density distribution in the form of a surface map is a convenient tool for visual evaluation of the results obtained for different groups of patients in comparison with the traditional point-to-point assessment of the relationship or average assessment of association.

Averaging for each group was carried out for means, $\left\langle {{\text{WB}}_{{{\text{EEG}}_{i} ,{\text{EEG}}_{j} }}^{{\Delta f_{k} }} } \right\rangle$:

4 $$\left\langle {{\text{WB}}_{{{\text{EEG}}_{i} ,{\text{EEG}}_{j} }}^{{\Delta f_{k} }} } \right\rangle_{1,2} = \mathop \sum \nolimits_{r = 1}^{{P_{1,2} }} \left\langle {{\text{WB}}_{{{\text{EEG}}_{i} ,{\text{EEG}}_{j} }}^{{\Delta f_{k} }} } \right\rangle_{1,2} /P,$$

where *P* corresponds to the number of points in the EEG records during the full duration of the first and second nights or during certain time intervals of the record corresponding to certain sleep stages on hypnograms. Performed calculations allowed assessing the functional connectivity strength for different frequency bands from EEG data, thereby assessing the spatial structure of the relationship between slow and fast oscillatory processes in the brain electrical activity.

To compare the synchronization levels between the first and second nights, we calculated $\left. {\Delta \left( {\left\langle {{\text{WB}}_{{{\text{EEG}}_{i} ,{\text{EEG}}_{j} }}^{{\Delta f_{k} }} } \right\rangle } \right)} \right|_{1 - 2}$ as:

5 $$\left. {\Delta \left( {\left\langle {{\text{WB}}_{{{\text{EEG}}_{i} ,{\text{EEG}}_{j} }}^{{\Delta f_{k} }} } \right\rangle } \right)} \right|_{1 - 2} = \frac{{\left\langle {{\text{WB}}_{{{\text{EEG}}_{i} ,{\text{EEG}}_{j} }}^{{\Delta f_{k} }} } \right\rangle_{1} - \left\langle {{\text{WB}}_{{{\text{EEG}}_{i} ,{\text{EEG}}_{j} }}^{{\Delta f_{k} }} } \right\rangle_{2} }}{{\left\langle {{\text{WB}}_{{{\text{EEG}}_{i} ,{\text{EEG}}_{j} }}^{{\Delta f_{k} }} } \right\rangle_{1} + \left\langle {{\text{WB}}_{{{\text{EEG}}_{i} ,{\text{EEG}}_{j} }}^{{\Delta f_{k} }} } \right\rangle_{2} }}$$

To track changes in the connectivity strength, based on EEG, during different sleep stages, we conducted pairwise evaluation of the following comparative characteristics:

6 $$\left. {\Delta \left( {\left\langle {{\text{WB}}_{{{\text{EEG}}_{i} ,{\text{EEG}}_{j} }}^{{\Delta f_{k} }} } \right\rangle } \right)} \right|_{{{\text{stage}} - 1,2}} = \frac{{\left\langle {{\text{WB}}_{{{\text{EEG}}_{i} ,{\text{EEG}}_{j} }}^{{\Delta f_{k} }} } \right\rangle_{{{\text{stage}}}} - \left\langle {{\text{WB}}_{{{\text{EEG}}_{i} ,{\text{EEG}}_{j} }}^{{\Delta f_{k} }} } \right\rangle_{1,2} }}{{\left\langle {{\text{WB}}_{{{\text{EEG}}_{i} ,{\text{EEG}}_{j} }}^{{\Delta f_{k} }} } \right\rangle_{{{\text{stage}}}} + \left\langle {{\text{WB}}_{{{\text{EEG}}_{i} ,{\text{EEG}}_{j} }}^{{\Delta f_{k} }} } \right\rangle_{1,2} }}.$$

### Statistical data processing

Mean, median, range deviation, quartile deviation, and standard deviation were used in descriptive statistics of the data. To compare quantitative data, the Mann–Whitney U test for independent samples was used^64,65^. Results with *p*-values ≤ 0.005 and ≤ 0.0005 were considered statistically significant. Statistical data processing was performed using STATISTICA version 10.0 for Windows (StatSoft Inc., Tulsa, Oklahoma, USA).

## Supplementary Information

Supplementary Information.

## Data Availability

The datasets generated and analyzed in the course of our study are available on reasonable request from Dr. Thomas Penzel (thomas.penzel@charite.de). The data are not publicly available due to presumed privacy or ethical restrictions.
