# Supplementary material for: Compensatory mechanisms of reduced interhemispheric EEG connectivity during sleep in patients with apnea
Source: Sci Rep. 2023 May 25;13:8444. doi: 10.1038/s41598-023-35376-1 (PMC10213009; doi:10.1038/s41598-023-35376-1)
Supplement: Supplementary file 1 — Supplementary Information. [file 41598_2023_35376_MOESM1_ESM.pdf]

Appendix I

Diagrams of distributions of $\Delta\left(\langle \mathrm{WB}^{\Delta f_k}_{\mathrm{EEG}_i,\mathrm{EEG}_j}\rangle\right)\Big|_{\mathrm{stage}-1,2}$, calculated for stages N1, N2, N3 of slow-wave and REM sleep, as well as selected periods of nocturnal wakefulness.

A pair of channels for which the calculation was performed is indicated for each set of diagrams. The gray background designates the range of differences in synchronization estimates within [-0.1; 0.1] for the first and second nights. Calculation results for groups I and II are coded with blue and red, correspondingly.

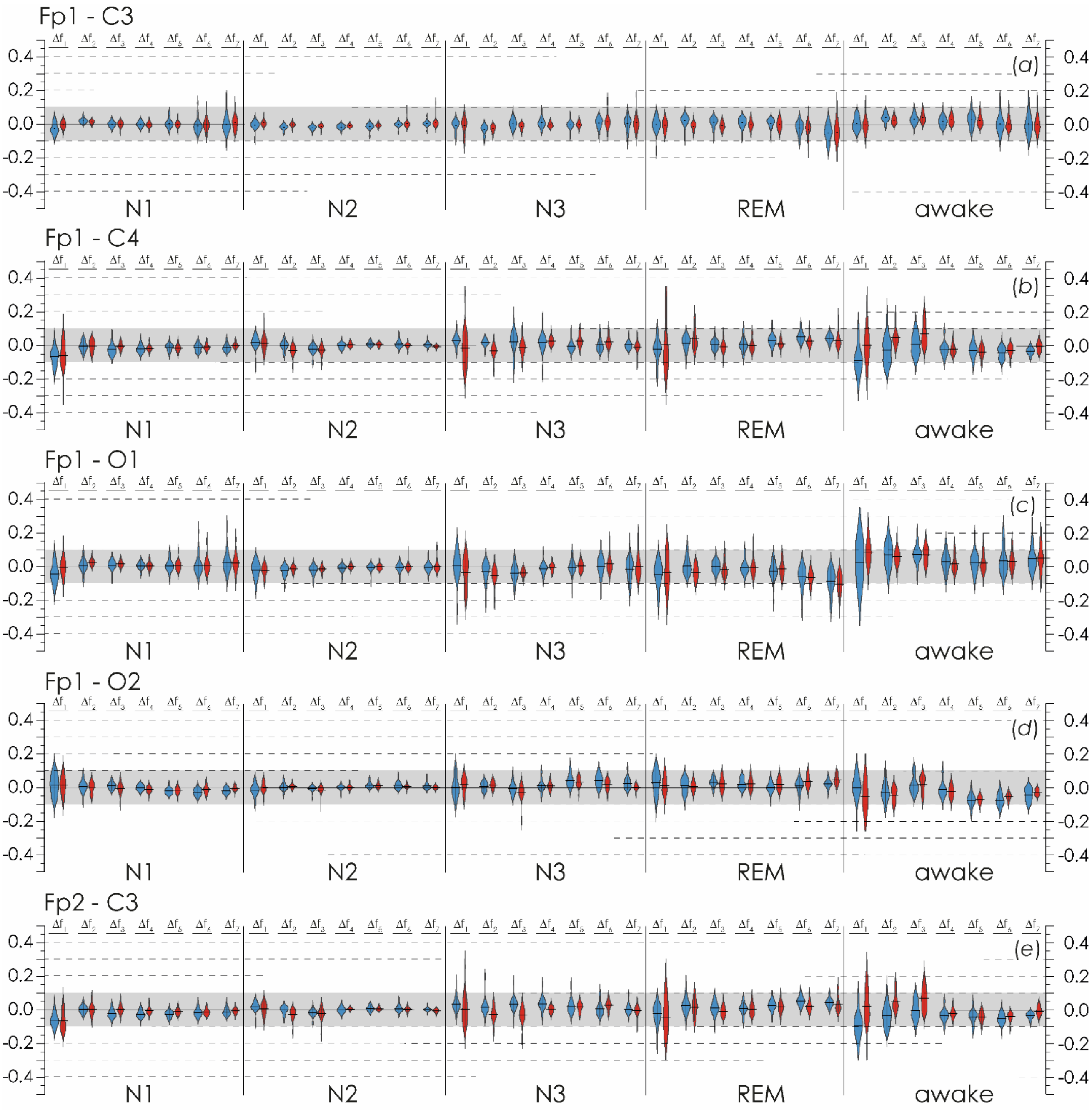

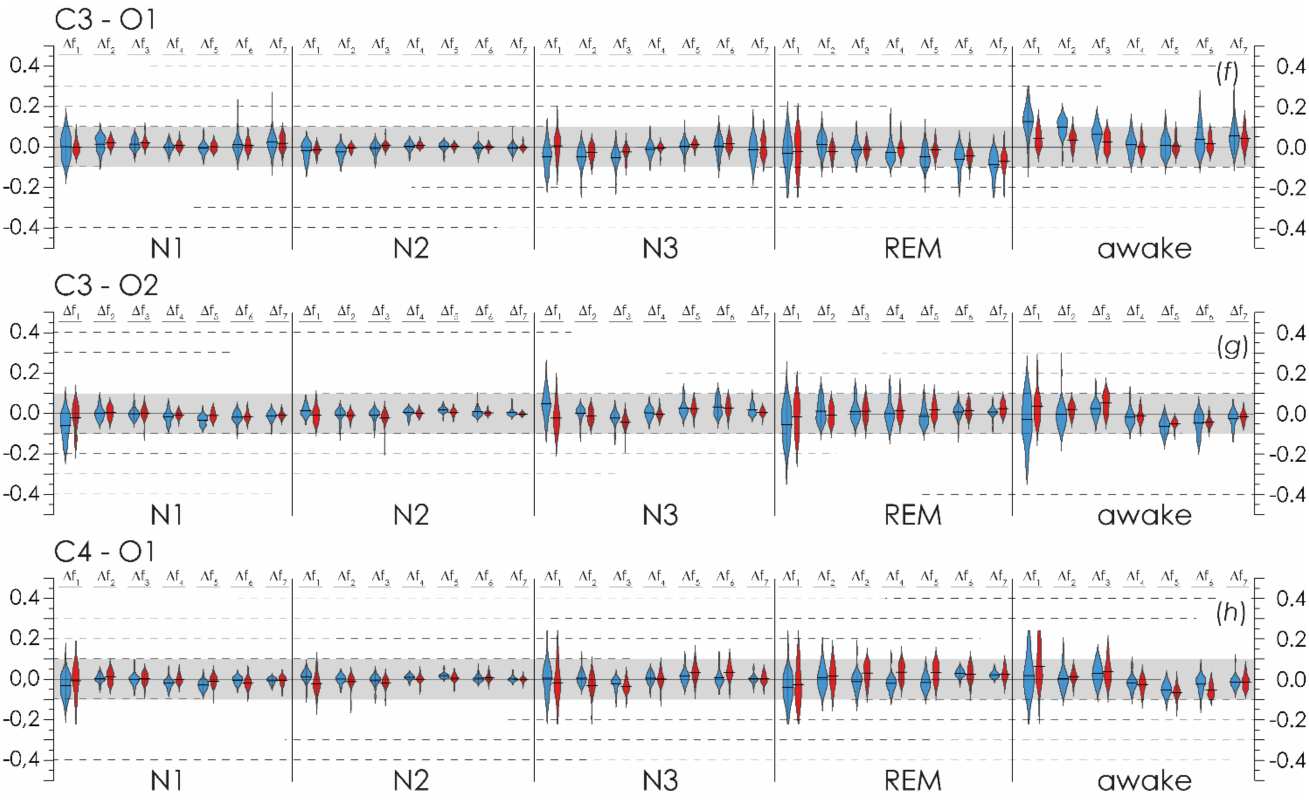
C3 - O1
(f)
C3 - O2
(g)
C4 - O1
(h)
N1
N2
N3
REM
awake
Δf1 Δf2 Δf3 Δf4 Δf5 Δf6 Δf7
0.4
0.2
0.0
-0.2
-0.4

Appendix II: Group analysis of changes in the synchronization strength of interhemispheric interactions

For ease of interpretation, the tables below use a separate color scale for each column of numerical data. Values exceeding the median are shown in green, while values less than the median are shown in red. The intensity of a particular color corresponds to the magnitude of a deviation from the median value in the column of numerical data.

Group analysis of changes in the synchronization strength between occipital EEG channels, O1 and O2

Frequency band	Group	Range deviation	Quartile range deviation	Standard deviation
Δf_1	Group I	0.52613	0.16084	0.12844
	Group II	0.30049	0.06715	0.06594
Δf_2	Group I	0.48882	0.21247	0.14025
	Group II	0.20605	0.04487	0.05996
Δf_3	Group I	0.4522	0.18662	0.13845
	Group II	0.22864	0.05075	0.06499
Δf_4	Group I	0.39381	0.17683	0.10884
	Group II	0.22308	0.03675	0.06008
Δf_5	Group I	0.36019	0.12934	0.09403
	Group II	0.2518	0.0645	0.06999
Δf_6	Group I	0.30124	0.09937	0.07517
	Group II	0.17238	0.07381	0.04916

Group analysis of changes in the synchronization strength between occipital EEG channels, C3 and C4

Frequency band	Group	Range deviation	Quartile range deviation	Standard deviation
Δf_1	Group I	0.2626	0.06005	0.06086
	Group II	0.32822	0.14076	0.0933
Δf_2	Group I	0.41662	0.13916	0.11382
	Group II	0.20257	0.10631	0.05595
Δf_3	Group I	0.42708	0.17003	0.11745
	Group II	0.15353	0.04961	0.03992
Δf_4	Group I	0.35117	0.14951	0.09361
	Group II	0.07226	0.04294	0.02299
Δf_5	Group I	0.29808	0.09342	0.07143
	Group II	0.12252	0.03403	0.0339
Δf_6	Group I	0.16124	0.04429	0.0409
	Group II	0.12386	0.03015	0.03503

Group analysis of changes in the synchronization strength between occipital EEG channels, Fp1 and Fp2

Frequency band	Group	Range deviation	Quartile range deviation	Standard deviation
Δf_1	Group I	0.3103	0.07067	0.07538
	Group II	0.27286	0.08848	0.07083
Δf_2	Group I	0.44364	0.15295	0.11646
	Group II	0.28097	0.10926	0.08444
Δf_3	Group I	0.47144	0.15672	0.11426
	Group II	0.25081	0.09646	0.06729
Δf_4	Group I	0.37381	0.11205	0.09315
	Group II	0.15757	0.09229	0.04877
Δf_5	Group I	0.31466	0.11333	0.07743
	Group II	0.21994	0.07543	0.05796
Δf_6	Group I	0.28095	0.11716	0.06737
	Group II	0.19578	0.06363	0.05155

Appendix III: Clinical characteristics of patients and their polysomnographic records

The following abbreviations are used in the Tables:

Gender (F/M): F = female; M = male

BPsys/BPdia = systolic blood pressure / diastolic blood pressure

ODI = oxygen desaturation index

N_{Ap} = number of apneic episodes

N_{Hyp} = number of hypopneic episodes

TST = total sleep time

AI = apnea index for TST

HI = hypopnea index for TST

AHI = apnea-hypopnea index for TST

Group I: apparently healthy patients

Number of patients, Np	Age, years	Gender	Height, cm	Weight, kg	Pulse	BPsys/BPdia	Number of records, Nr	ODI
1	51	M	164	60	80	95/60	1	0.0
							2	0.3
2	22	M	174	66	50	110/70	1	0.2
							2	0.2
3	41	F	164	53	100	120/80	1	0.4
							2	1.1
4	26	F	176	70	80	90/60	1	0.4
							2	0.2
5	33	F	158	70	80	110/80	1	0.2
							2	1.0
6	29	M	187	91	76	90/60	1	0.2
							2	0.4
7	50	M	165	60	84	130/90	1	0.3
							2	0.3
8	35	M	183	68	72	120/90	1	0.5
							2	0.4
9	50	F	161	64	76	120/90	1	0.6
							2	0.4
10	34	M	174	93	76	120/80	1	0.2
							2	0.4
11	76	F	166	70	84	160/90	1	0.0
							2	0.0
12	63	M	173	80	82	120/70	1	0.0
							2	0.0
13	72	F	158	65	84	145/80	1	0.5
							2	0.5
14	81	F	158	88	84	150/84	1	0.0
							2	0.0
15	42	M	169	76	80	130/80	1	0.4
							2	1.3

Group II: patients with sleep apnea syndrome

Np	Age, years	Gender	Height, cm	Weight, kg	Pulse	BPsys/BPdia	Nr	ODI	N_{Ap}	N_{Hyp}	AI	HI	AHI
16	48	M	175	82	72	160/100	1	30.1	331	36	50.923	5.5	56.5
							2	39.6	312	45	44.099	6.4	50.5
17	49	F	158	73	80	140/90	1	6.5	7	91	1.083	14.0	15.2
							2	5.3	32	123	4.588	18.0	22.2
18	41	M	177	80	74	146/92	1	0.4	6	44	1.214	8.9	10.1
							2	2.8	99	104	14.488	15.0	29.7
19	67	F	167	88	68	160/104	1	13.2	80	46	14.118	8.1	22.2
							2	17	105	72	14.189	9.7	23.9
20	47	F	170	110	76	126/86	1	9.3	76	214	10.471	29.0	40.0
							2	13.1	97	222	12.694	29.0	41.7
21	62	M	165	75	76	140/98	1	2.0	2	9	0.333	1.5	1.83
							2	0.5	11	125	1.575	18.0	19.5
22	58	M	176	83	64	150/100	1	2.7	35	168	6.472	31.0	37.5
							2	5.5	62	108	10.136	18.0	27.8
23	59	M	185	82	59	154/94	1	8.4	67	33	9.951	4.9	14.9
							2	5.3	42	43	5.701	5.8	11.5
24	53	M	168	80	75	150/95	1	39.2	404	49	69.760	8.5	78.2
							2	29.1	335	6	51.600	0.9	52.5
25	44	M	177	80	75	120/80	1	13.7	24	116	3.450	17.0	20.1
							2	12.3	88	68	11.200	8.7	19.9

We calculated the complex valued wavelet coefficients $W_i(f, t_0)$ for each EEG channel $EEG_i(t)$ as:

$$W_i(f,t_0) = \sqrt{f} \cdot \int_{t_0-4/f}^{t_0+4/f} \mathrm{EEG}_i(t) \cdot \psi^*(f,t-t_0) \cdot dt, \quad (3)$$

where $i = 1, \ldots, 6$ is the number of considered EEG channel, t_0 specifies the wavelet location on the time axis, "*" denotes the complex conjugate, and $\psi^*(f,t)$ is the mother wavelet function. We used the standard Morlet wavelet, which is often employed for processing of biological signals [33]:

$$\psi(f,t-t_0) = \sqrt{f} \cdot \pi^{-1/4} \cdot exp(\imath \cdot \omega_0 \cdot f \cdot (t-t_0)) \cdot exp\left(-f^2 \cdot {(t-t_0)^2}/{2}\right), \quad (4)$$

where ω_0 is the wavelet scaling parameter and $\imath$ is an imaginary unit. It is well known that the parameter $\omega_0 = 2\pi$ provides an optimal time-frequency resolution of the EEG signal in the continuous wavelet transformation (CWT) [38, 39]. To measure the degree of coherence between two EEG signals, $EEG_i(t)$ and $EEG_j(t)$, we used the corresponding complex valued wavelet coefficients $W_i(f, t) = a_i + \imath\, b_i$ and $W_j(f, t) = a_j + \imath\, b_j$.

Wavelet bicoherence, $\mathrm{WB}_{ij}(f, t)$, is estimated based on the mutual wavelet spectrum $W_{i,j}(f, t)$$ of the signals $EEG_i(t)$ and $EEG_j(t)$. Similarly to [15, 40], the coefficients Re $[\mathrm{WB}_{ij}(f, t)]$ and Im $[\mathrm{WB}_{ij}(f, t)]$, presented as real and imaginary parts of mutual wavelet spectrum, can be calculated as:

$$Re[\mathrm{WB}_{i,j}(f,t)] = \frac{a_i(f,t)\cdot a_j(f,t)+b_i(f,t)\cdot b_j(f,t)}{\sqrt{a_i^2(f,t)+b_i^2(f,t)}\cdot\sqrt{a_i^2(f,t)+b_i^2(f,t)}}, \quad (5)$$

and

$$Im[\mathrm{WB}\tau_{i,j}(f,t)] = \frac{b_i(f,t)\cdot a_j(f,t)-a_i(f,t)\cdot b_j(f,t)}{\sqrt{a_i^2(f,t)+b_i^2(f,t)}\cdot\sqrt{a_i^2(f,t)+b_i^2(f,t)}}. \quad (6)$$

Thus, the synchronization value of two EEG channels, $EEG_i(t)$ and $EEG_j(t)$, at frequency f is calculated as follows:

$$\mathrm{WB}_{i,j}(f,t) = \sqrt{\left(Re[\mathrm{WB}_{i,j}(f,t)]\right)^2 + \left(Im[\mathrm{WB}_{i,j}(f,t)]\right)^2}. \quad (7)$$
